# Complete chloroplast genome sequence of a subtropical tree, *Parasassafras confertiflorum* (Lauranceae)

**DOI:** 10.1080/23802359.2018.1532331

**Published:** 2018-10-31

**Authors:** Qiong Liao, Tinghong Ye, Yu Song

**Affiliations:** a State Key Laboratory of Biotherapy, West China Hospital, Sichuan University, Chengdu, Sichuan, China;; b Center for Integrative Conservation, Xishuangbanna Tropical Botanical Garden, Chinese Academy of Sciences, Mengla, China;; c Southeast Asia Biodiversity Research Institute, Chinese Academy of Science, Yezin, Nay Pyi Taw, Myanmar

**Keywords:** *Parasassafras confertiflorum*, Lauraceae, chloroplast genome, phylogenetic analyses

## Abstract

*Parasassafras confertiflorum* (Meisn.) D.G. Long is the single tree species of the genus *Parasassafras* D.G. Long in the family Lauraceae. To better determine its phylogenetic location with respect to the related Lauraceae species, the complete chloroplast genome of *P. confertiflorum* was sequenced. The whole plastome is 152,555 bp in length, consisting of a pair of inverted repeat (IR) regions of 20,079 bp, one large single copy (LSC) region of 93,604 bp, and one small single copy (SSC) region of 18,793 bp. The genome contains 127 genes, including 83 protein-coding genes, 8 ribosomal RNA genes, and 36 transfer RNA genes. The overall GC content of the whole plastome is 39.1%. Further, maximum likelihoodphylogenetic analyses were conducted using 34 complete plastomes of the Lauraceae, which support close relationships between *P. confertiflorum* and *Actinodaphne trichocarpa*, *Lindera benzoin*, *L. latifolia*, *L. metcalfiana*, *L. robusta*, and *Neolitsea sericea* rather than *Laurus nobilis* or *Sinosassafras flavinervium*.

The genus *Parasassafras* D.G. Long in the family Lauraceae includes only one species *Parasassafras confertiflorum* (Meisn.) D.G. Long rarely distributed at high altitudes in Yunnan of SW China, Bhutan, India, and Myanmar (Huang and Henk 2008). For a better understanding of the relationships of *P. confertiflorum* and its related species, it is necessary to reconstruct a robust phylogenetic tree of the core Laureae group based on high-throughput sequencing approaches.

Young leaves of *P. confertiflorum* in Ximeng County (Yunnan, China; Long. 99.6687 E, Lat. 22.7615 N, 1788 m) were picked for DNA extraction (Doyle and Dickson 1987). The voucher was deposited at the Biodiversity Research Group of Xishuangbanna Tropical Botanical Garden (Accession Number: XTBG-BRG-SY34285). The whole chloroplast genome was sequenced following Zhang et al. (2016), and their 15 universal primer pairs were used to perform long-range PCR for next-generation sequencing. The contigs were aligned using the publicly available cp genome of *Lindera glauca* (GenBank accession number MF188124) and annotated in Geneious 4.8.

The plastome of *P. confertiflorum* (GenBank accession number MH729378.), with a length of 152,555 bp, was 65 bp and195 bp smaller than that of *Litsea glutinosa* (152,618 bp, KU382356) and *Laurus nobilis* (152,750 bp, KY085912). It was also 7 bp and 113 bp larger than that of the *Lindera benzoin* (152,478 bp, MH220730) and *Neolitsea sericea* (152,442 bp, MF939341) (Hinsinger and Strijk 2017; Song et al. 2017a; Zhao et al. 2018). The lengths of the inverted repeats (IRs), small single-copy (SSR) region, and large single-copy (LSC) region in the plastome of *P. confertiflorum* was 20,079 bp, 18,793 bp, and 93,604 bp, respectively. The G + C contents of the plastome is 39.1%.

The complete plastid genome sequence of *P. confertiflorum* and the other sequenced Lauraceae taxa including *Actinodaphne trichocarpa*, *Alseodaphne gracilis*, *A. huanglianshanensis*, *A. semecarpifolia*, *Cinnamomum camphora*, *C. kanehirae*, *C. micranthum*, *C. verum*, *Laurus nobilis*, *Lindera benzoin*, *L. communis*, *L. glauca*, *L. latifolia*, *L. megaphylla*, *L. metcalfiana*, *L. nacusua*, *L. obtusiloba*, *L. robusta*, *Litsea glutinosa*, *Machilus balansae*, *M. pauhoi*, *M. thunbergii*, *M. yunnanensis*, *Nectandra angustifolia*, *Neolitsea sericea*, *Persea americana*, *Phoebe chekiangensis*, *P. omeiensis*, *P. sheareri*, *P. zhennan*, and *Sassafras tzumu* (Song et al. 2015, 2016, 2017a, 2017b, 2018; Hinsinger and Strijk 2017; Wu et al. 2017; Zhao et al. 2018), formed the base to perform a phylogenetic analysis, with *Endiandra globosa* and *E. discolor* as outgroup. A maximum likelihood analysis (Tamura et al. 2011) yielded a tree topology with 39–100% bootstrap (BS) values at each node. The topology divided the 32 Lauraceae taxa into three 100% supported clades (Figure 1). The first divergent clade contained *Alseodaphne*, *Machilus*, *Persea*, and *Phoebe* taxa, the second *Cinnamomum*, *Nectandra*, and *Sassafras* taxa, and the third contained *Actinodaphne*, *Laurus*, *Lindera*, *Litsea*, *Neolitsea* species and *P. confertiflorum*. This phylogenetic tree reveals that *Lindera* is polyphyletic and there are close relationships between *P. confertiflorum* and *Actinodaphne trichocarpa*, *Lindera benzoin*, *L. latifolia*, *L. metcalfiana*, *L. robusta*, and *Neolitsea sericea* rather than *Laurus nobilis* or *Sinosassafras flavinervium*.

**Figure 1.**
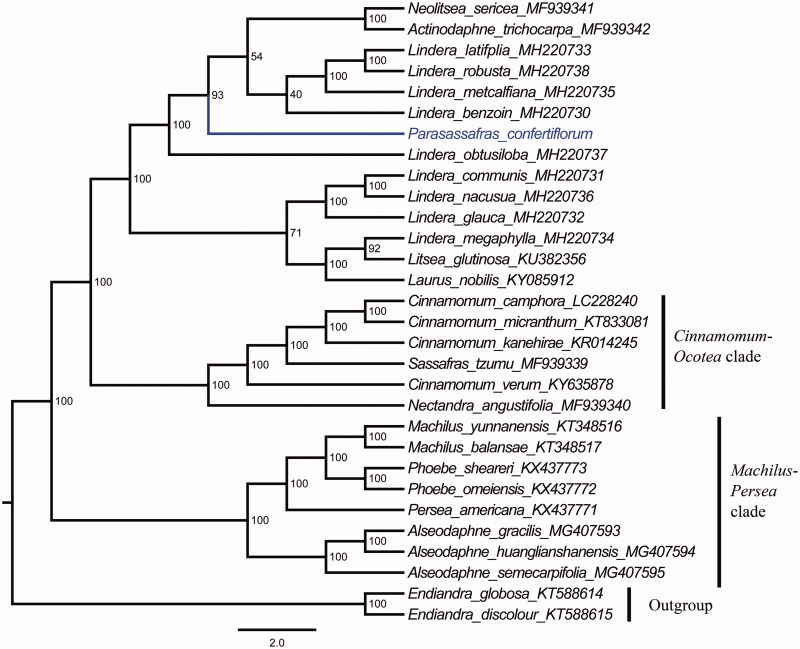
Molecular phylogenetic tree of 34 taxa of Lauranceae based on complete plastome sequences using unpartitioned ML. Number at each node are bootstrap support value.
